# Mental- and physical health, and general well-being in patients with polyposis syndromes: a scoping review

**DOI:** 10.1007/s10689-026-00537-4

**Published:** 2026-02-14

**Authors:** Sophie Walton Bernstedt, Anna Jervaeus, Veronica Höiom, Kaisa Fritzell

**Affiliations:** 1 Division of Clinical Medicine, Department of Medicine Huddinge, Karolinska Institutet, Stockholm, Sweden; 2 Division of Gastroenterology, Dermatology and Rheumatology, Karolinska University Hospital, Stockholm, Sweden; 3 Division of Upper Gastrointestinal Diseases, Karolinska University Hospital, Stockholm, Sweden; 4 Division of Nursing, Department of Neurobiology, Care Sciences and Society, Karolinska Institutet, Stockholm, Sweden; 5 Department of Oncology and Pathology, Karolinska Institutet, Stockholm, Sweden; 6 Hereditary Cancer Outpatient Clinic, Cancer Theme, Karolinska University Hospital, Stockholm, Sweden

**Keywords:** Familial adenomatous polyposis, Peutz jeghers syndrome, Psychological well-being, QOL

## Abstract

Polyposis syndromes are associated with an increased lifelong cancer risk in several organs as well as a commitment to regular examinations and risk-reducing surgery of healthy organs, which can be compared to living with a constant health threat. More recently conducted research on the topic is scarce, and, therefore, this scoping review synthesized data of existing research on mental and physical health and general well-being from the patients’ perspectives. The Preferred Reporting Items for Systematic Reviews and Meta-Analyses Extension for Scoping Reviews (PRISMA-ScR) was used. The search was conducted between January 2000 and October 2024, leading to the identification of 4138 studies. Of those, 39 studies met the inclusion criteria (1%), and of the 2214 included participants, 2108 had familial adenomatous polyposis. In order to analyze and synthesize relevant data, aspects of directed content analysis were applied. The findings show that patients exhibited mental and physical health in parity to population norms. However, feelings of vulnerability, anxiety, worry and fear, especially in younger participants were reported. In addition, decreased bowel and sexual function, food restrictions, body dissatisfaction, and physical attractiveness were evident. Regarding well-being, an altered lifestyle, work and social discrimination, insurance matters, concerns for family members and fertility were reported. Being female seemed to be related to mental health issues or vulnerability thereof. Appropriate screening tools, such as patient reported outcome measures (PROM), should be developed and implemented in a structured way to capture patients needs in clinical settings. Future research is needed for the rarer syndromes, represented to a limited extent in this review.

**Supplementary Information:**

The online version contains supplementary material available at 10.1007/s10689-026-00537-4.

## Introduction

In polyposis syndromes, such as Familial adenomatous polyposis (FAP) and Peutz Jeghers syndrome (PJS), an underlying hereditary factor (pathogenic variant) is present. Polyposis syndromes represent only 2% of all colorectal cancer cases in Sweden, but the syndromes are associated with a high risk of cancer [1]. The syndromes share similarities, such as conferring an increased lifetime risk of bowel cancer (originating from polyps), although age of onset differs. Since these conditions are genetic syndromes, there is also an increased lifetime risk of other organ cancer. To ensure early detection and to prevent cancer, these individuals need to adhere to lifelong surveillance programs, including regular endoscopic examinations, as well as risk-reducing surgery of healthy organs. Both FAP and PJS are inherited in an autosomal dominant way, which means that there is a 50% risk of transferring the pathogenic variant to offspring.

## Background

FAP is caused by pathogenic variants in the *APC* gene. The lifetime risk of colorectal cancer is estimated to be 100%. To reduce the cancer risk, removal of the colon is recommended, followed by endoscopic examinations, for some as often as twice a year. In addition, individuals with FAP have an increased risk of duodenal cancer and are therefore recommended further endoscopic examinations, and risk-reducing removal of the duodenum is often performed. Reported rates of duodenectomy in patients with FAP range between 5.5 and 8.2% [1, 2]. Moreover, there is also an increased risk of desmoid tumors. Desmoid tumors are benign but can grow aggressively. Pre-symptomatic genetic testing of offspring regarding the pathogenic variant identified in a parent is recommended at the ages of 12–18 years [3]. PJS is caused by pathogenic variants in the gene *STK11*. Enlarged polyps causing intussusception and anemia due to bleeding are also morbidities related to PJS, and the lifetime bowel cancer risk is estimated to be about 50–60% [4]. In addition, an increased lifetime cancer risk exists in several other organs such as breasts, testicles, lungs, pancreas, and the gynecological organs. To prevent morbidity related to enlarged polyps and cancer, regular endoscopic examinations once a year are recommended, as well as regular mammography, ultrasounds, and magnetic resonance imaging [5]. Genetic tests of offspring are recommended at the ages of 10–12 years.

Living with a polyposis syndrome is associated with an increased lifelong cancer risk in several organs. This, together with commitment to regular examinations of several organs, can be compared to living with a constant health threat and a worry for offspring. A systematic review from 2012 [6] aimed at investigating psychological aspects of participating in surveillance programs for a variety of hereditary cancer syndromes, including PJS and FAP. The findings included various results regarding quality of life and distress. Conclusions made from the review were that factors such as cancer worry, lack of social support, young age, female gender, being single, and lower educational level were associated with increased distress or a poorer quality of life. Moreover, examinations were reported as painful and uncomfortable, although most patients perceived benefits from the surveillance program. The period of genetic investigation can be especially stressful, including the risk of transferring the pathogenic variant to offspring and worry for the health of family members [6].

Polyposis syndromes are relatively rare and therefore encumbered with certain challengesrelated to the limited number of specialist clinics and well-educated healthcare staff. A Swedish study on patients with FAP, conducted by one of the authors (KF), found that patients receiving care at a specialist clinic with a multidisciplinary team reported significantly better quality of care, including the possibility to participate in care, compared with patients cared for at non-specialist clinics [7]. Moreover, building collaborative relationships with family physicians were reported by parents with polyposis syndromes to be very important for their ability to cope with their child’s health condition. In addition, building reassuring relationships with other parents in the same situation and sharing experiences could mitigate feelings of uncertainty and anxiety and increase coping abilities [8]. Meeting the needs of patients in Sweden is particularly challenging due to the scarcity of specialist clinics, the absence of centralized care, and the occasionally lengthy travel distances.

To the best of our knowledge, more recently conducted research, especially reviews, on the topic is scarce, and in order to properly design and develop appropriate health care and support for individuals affected by polyposis syndromes more knowledge and insight are needed on how their lives are affected. Therefore, the aim of this scoping review was to describe mental and physical health and general well-being, from the patients’ perspectives, when living with a high risk of cancer caused by a polyposis syndrome.

## Methods

### Design

To synthesize evidence on mental and physical health and well-being in individuals with polyposis syndromes, a scoping review, i.e., a review descriptive by nature, was conducted [9]. We used the description of well-being stated by the World Health Organization (WHO): a resource for daily life determined by social, economic, and environmental aspects important for the ability to contribute to the world with a sense of meaning and purpose [10]. Results were reported according to The Preferred Reporting Items for Systematic Reviews and Meta-Analyses Extension for Scoping Reviews (PRISMA-ScR) in order to suitably map available studies according to the objective [9, 11]**.**

### Information sources and searches

A literature search was performed in the following databases: Medline, Embase, Web of Science, and Cinahl. The original search was performed on 2024-04-17and last updated on 2024-10-22 by rerunning the searches and deduplicating against previous results, using Covidence [12]. The search strategy was developed in Medline (Ovid) in collaboration with librarians at the Karolinska Institutet University Library. For each search concept, Medical Subject Headings (MeSH terms) and free text terms were identified. The strategies were peer-reviewed by another librarian prior to execution. The search was then translated, in part using Polyglot Search Translator [13], into the other databases. No language restriction was applied. Databases were searched during the period 2000 to 2024. De-duplication was done using the method described by Bramer et al. [12]. One final extra step was added to compare DOIs. The search strategy resulted in 4138 titles. In addition, a manual search (PubMed, Web of Science, Google) was applied to check for additional studies relevant for this scoping review. The manual searches resulted in 10 more titles. The full search strategies for all databases are available as a supplementary file. For inclusion and exclusion criteria, see Table 1.

**Table 1:** Inclusion and exclusion criteria’s

*Inclusion criteria*
Must include patients with verified FAP^a^, MAP^b^, JP^c^ and PJS^d^
Must discuss the impact on mental/psychological health and/or physical health and/or well-being, patient perspective
*Exclusion criteria*
Mixed samples with a minority of FAP^a^, MAP^b^, JP^c^ and PJS^d^ patients
Mixed sample where results specific for FAP^a^, MAP^b^, JP^c^ and PJS^d^ cannot be distinguished
Studies on healthcare perspectives or cost efficiency
FAP abbreviation not Familial adenomatous polyposis
No full text available
Non original research
Conference abstract

^a^FAP = familial adenomatous polyposis, ^b^MAP = MUTYH associated polyposis, ^c^JP = Juvenile polyposis, ^d^PJS = Peutz Jeghers syndrome

### Selecting sources of evidence and data charting

The selecting sources process aimed to identify key characteristics of each study as well as relevant information according to the aim. This process was performed in the program Rayyan [14]. Two reviewers (VH and KF) independently scrutinised the titles and abstracts in Rayyan, and inconsistencies were resolved through discussion with each other. Studies were selected based on the following inclusion criteria: 1/mental/psychological health—including sadness, depression, worry, guilt, psychological illness, and catastrophic thoughts; 2/physical health—including bowel symptoms, sexual function, and body image; 3/well-being—social life, work life, relationships, and family life. A data charting table with variables such as authors, publication year, country of origin, aim, study design, sample size, mean age, gender, type of polyposis syndrome, type of evidence source, and relevant outcomes was compiled (supplementary file).

Aspects of direct content analysis were applied to analyze and synthesize relevant data from articles. Directed content analysis is appropriate when prior research exists but could benefit from additional description [15]. After having read and assessed all studies (KF, VH, AJ, SWB), a pattern was evident to the authors that articles could be organized into three different subgroupsrelating to the aim: 1/ genetic testing, 2/ life after surgery, and 3/ life with a polyposis syndrome. We applied the three inclusion criteria as research questions: 1/Does the polyposis syndrome influence mental health?, 2/Does the polyposis syndrome influence physical health?, 3/Does the polyposis syndrome influence general well-being? These questions were posed to the result sections in each article, and relevant information was extracted.

To keep track of the studies, the data charting table (Microsoft Excel ®) was used to document the analysis. Corresponding author (KF) and co-authors (VH, AJ, SWB) were responsible for extracting relevant information and writing summaries for one subgroup each (genetic testing VH; surgery SWB, KF; life AJ). The corresponding author had the main responsibility for the analytical process and compilation of results. The analytical process was then discussed among all authors until a final agreement was reached.

## Results

### Selection of sources of evidence

**Fig. 1:**
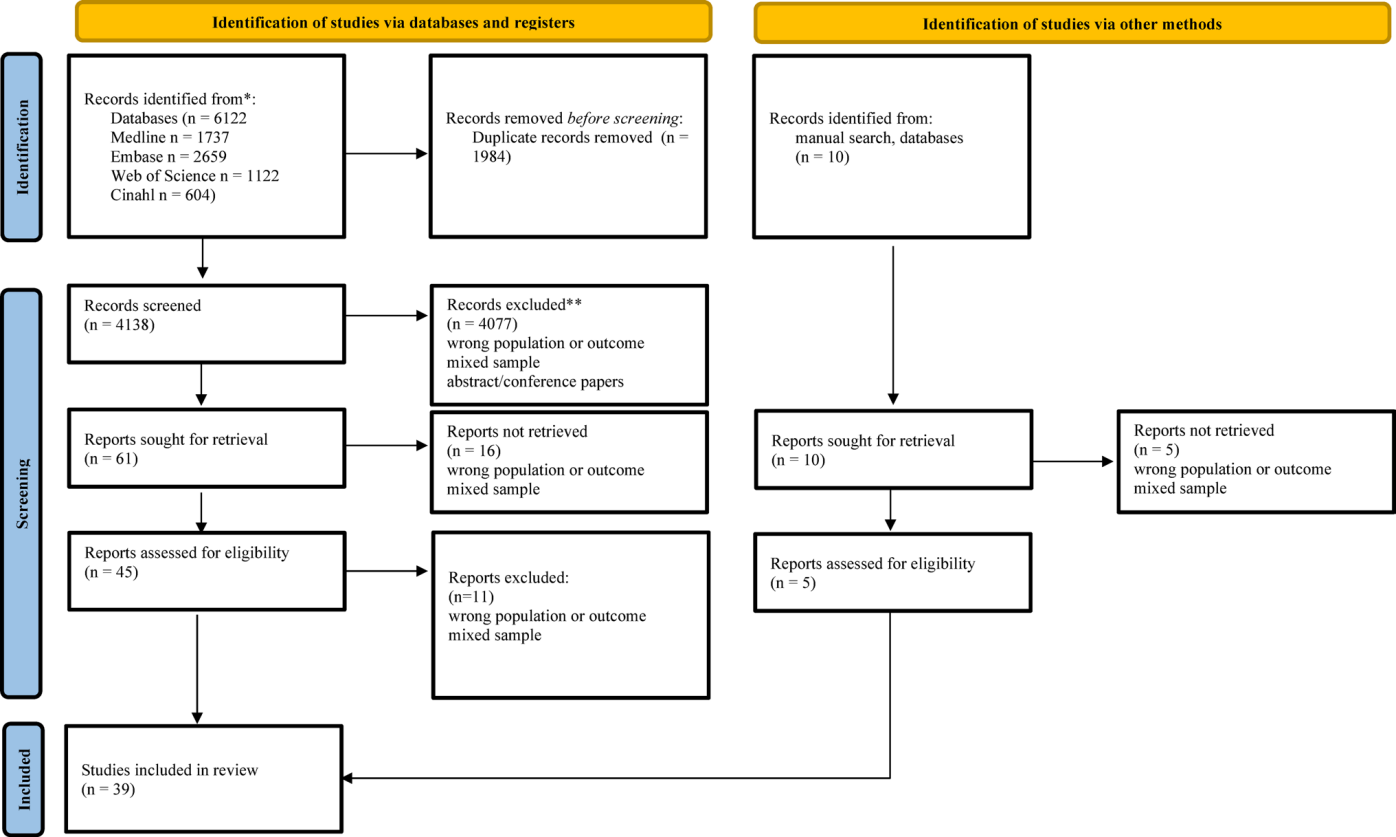
PRISMA 2020 flow diagram

The search strategy outlined was conducted up to October 2024, leading to the identification of 6122 titles. A total of 1984 duplicates were identified and removed, resulting in 4138 titles. These were screened by title and abstract for relevance, and 4077 were excluded. Forty-five studies were selected for full-text analysis, of those, 11 studies did not meet the inclusion criteria and were excluded. Ten additional titles were identified through manual searches, out of which five studies met the inclusion criteria (Fig. 1).

### Characteristics of sources of evidence

Of the 39 included studies, 35 were quantitative, three were qualitative, and one was mixed method. Most studies, 29/39 (72%), were published before 2013. The studies were conducted in thirteen different countries, with the USA as the most common (n = 10, 25%). Of 2280 included participants, the vast majority of participants had FAP (n = 2174, 95%). For more details, see Table 2 and the supplementary file.

**Table 2:** Characteristics of included studies, n = 39

Characteristics	n	References
Population	Participants	
FAP^a^	2108	[1–22, 25–30, 32–35, 37–39]
MAP^b^	**-**	
JP^c^	**-**	
PJS^d^	106	[24, 25, 32, 37]

Characteristics	n	References
Publication years		
2000–2013	29	[2, 3, 5, 8–11, 13–17, 19–31, 33, 36, 37, 39]
2014–2024	10	[1, 4, 6, 7, 12, 18, 32, 34, 35, 38, 39]

Characteristics	n	References
Countries	Articles	
Australia	3	[2, 3, 28]
Canada	3	[7, 11, 14]
China	1	[32]
France	2	[6, 29]
Germany	6	[17–19, 30, 35, 36]
India	1	[34]
Israel	1	[23]
The Netherlands	6	[8–10, 24, 25, 39]
Saudi Arabia	1	[1]
Sweden	3	[12, 15, 16]
UK	1	[4]
UK/Australia	1	[27]
US	10	[5, 13, 20–22, 26, 31, 33, 37, 38]

Characteristics	n	References
Type of study		
Qualitative, n = 3		
Cross-sectional	3	[15, 28, 32]
Quantitative, n = 35		
Cross-sectional	17	[1–3, 7, 9–12, 16, 19, 21, 24, 25, 29, 35, 37, 38]
Longitudinal	7	[5, 13, 17, 23, 26, 34, 36]
prospective	3	[18, 20, 27]
Retrospective	8	[4, 6, 8, 22, 30, 31, 33, 39]
Mixed method (qualitative/quantitative), n = 1	1	[14]

^a^FAP = familial adenomatous polyposis, ^b^MAP = MUTYH associated polyposis, ^c^JP = Juvenile polyposis, ^d^PJS = Peutz Jeghers syndrome

### Synthesis of results

**Fig. 2:**
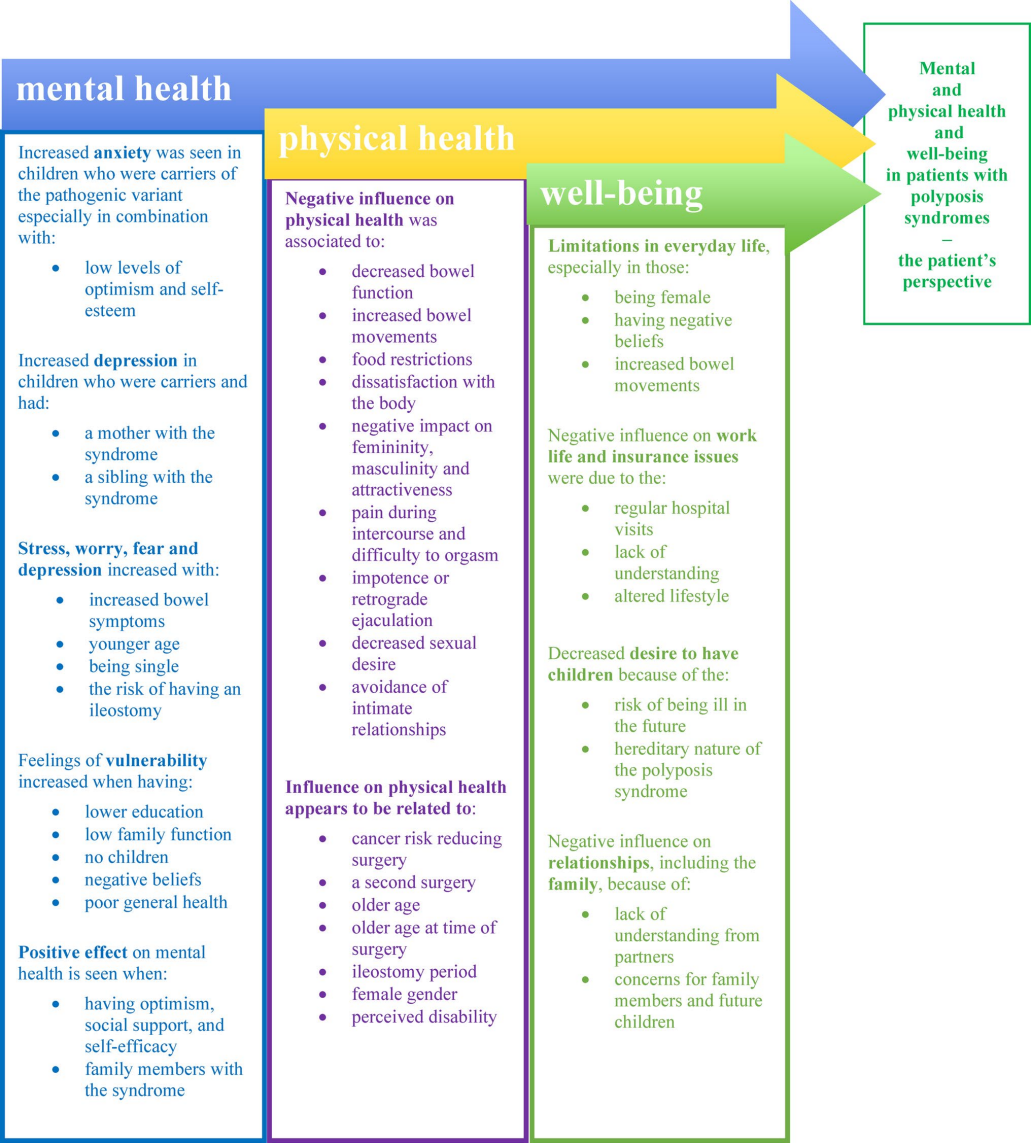
Synthesis and results regarding the three areas: mental health, physical health and well-being in individuals with polyposis syndromes

Findings of this scoping review were organised according to the aim and further according to the identified categories: genetic testing, the life after surgery, and the life with a polyposis syndrome (Table 3 and Fig. 2).

**Table 3:** Included studies organised according to identified categories

Category	Number	References or reference number?
Genetic testing	4	Andrews 2006, Codori 2010, Douma 2010, Michie 2001
Life after surgery	23	Andrews 2007, Christou 2016, Collard 2020, van Duijvendijk 2000, Durno 2012, Erkek 2007, Fritzell 2011, Ganschow 2010, Ganschow 2018, Gunther 2002, Hassan 2005, Ko 2000, Ko 2002, Krausz 2005, Lillehei 2010, Ortega-Deballon 2009, Osterfeld 2008, Ozdemir 2013, Parc 2000, Raviram 2015, Schneider 2015, Wolf 2011, de Zeeuw, 2011
Life with a polyposis syndrome	12	Alhassan 2024, Danieli 2024, Douma 2010, Eriksson 2016, Esplen 2004, Fritzell 2010, van Lier 2012, van Lier 2010, Mireskandari 2009, Pan 2024, Woo 2009, Wood 2019

### Mental health from the patients’ perspective

#### Genetic investigation

Evaluating long-term psychological effects of genetic testing for FAP, regarding both children and their parents, showed that depression symptom scores were within normal limits. Children who were carriers of the pathogenic variant and had a father who also was a carrier had lower depression scores than those whose mothers were carriers of the pathogenic variant. In addition, children who were carriers of the pathogenic variant with a sibling who also was a carrier had increased depression scores. Predictive genetic testing had a psychological impact in that children who were carriers of the pathogenic variant were worried to a higher extent and felt more threatened by their test result compared to those who did not carry the pathogenic variant. Clinical anxiety was seen in children who were carriers of the pathogenic variant, especially in combination with low levels of optimism and self-esteem. However, children were less anxious than adults in general [16, 17]

#### Life after surgery

In general, mental health, including anxiety and depression, in patients with FAP subjected to surgery was found to be in parity with population norms. Mental health seemed to be independent of the type of risk-reducing surgery of the colon, time since surgery, or an additional surgery of the duodenum. Optimism, social support, and self-efficacy were found to have a positive effect on mental health and exceeded those of the general population. However, FAP-specific emotional problems were reported, such as stress, worry, fear, depression and mental health was found to be predicted by increased bowel symptoms, being single, and younger age. Younger patients reported more distress surrounding surgery and worry about the cancer risk compared to older patients. Fear of having an ileostomy and future complications while waiting for risk-reducing surgery were also reported, and when having an ileostomy, patients reported disgust and shame. Mental health was found to remain stable or improve over time post-surgery. No differences were noted regarding psychological outcomes and self-esteem in patients with de novo FAP compared to those with a family history of FAP, although a family history of FAP positively correlated with avoidance, feelings of intrusion, and anxiety [18–26]

#### Life with a polyposis syndrome

Descriptions of uncertainty, unpredictability, feelings of isolation and loss were evident as well as embarrassment, worrying thoughts, distress, mild depression, guilt, reproductive stress, and fear of resentment. A worse outcome on social responsiveness was also evident when comparing participants with FAP to unaffected siblings. At the same time, on a group level, descriptions of similar distress, anxiety or depression scores as the general population were evident, as well as descriptions of being resilient and a wish to live life fully. Being female, having lower education, having no partner, low family functioning, no children, risk perception, negative beliefs, and poor general health seemed to be some factors related to mental health issues or vulnerability thereof [27–38]

## Physical health from the patients’ perspectives

### Genetic investigation

The result of the predictive genetic testing did not affect perceived physical health in children nor adults. Furthermore, the outcome of the test did not affect whether one regretted taking the test or not [17]

### Life after surgery

When using a standardised quality of life questionnaire, e.g., SF-36, physical health in patients with FAP was in all but one study in comparison with general populations. However, those studies that used a surgery-specific questionnaire to measure quality of life found that factors such as decreased bowel function, including increased daily and nocturnal bowel movements, perceived disability, female gender, and a second surgery influence physical health negatively. Those who were dissatisfied with their physical health perceived their life as worse after surgery, and the ileostomy period, especially, was perceived as worse than anticipated. Moreover, factors that had a negative influence on physical health and bowel function were older age at surgery and increasing age, although one study found that younger patients reported more incontinence and nocturnal stools and were more embarrassed due to bowel concerns than older patients [26, 39, 40, 42–44]

When comparing physical health between types of surgeries or surgery techniques, the results varied. Some studies reported no differences in physical health regarding bowel function, such as feacal frequency incontinence, in patients with ileo-anal pouch (IPAA), ileorectal anastomosis (IRA), and an additional duodenectomy, or in patients with restored bowel continuity IPAA, IRA or an ileostomy. In some studies, however, patients reported worse bowel function, including leakage, number of bowel movements, pad usage, perianal skin problems, food avoidance, and the inability to distinguish gas from stool when having an IPAA compared to having an IRA. When comparing surgery techniques, such as handsewn or stapled mucosectomy, both groups reported similar numbers of day- and nighttime bowel movements, although pad usage was higher in the mucosectomy group. One study did a long-term follow-up on ileo-neo-rectal anastomosis, an attempt to reduce procedure-related complications, found a perception of improved general health 2 and 7 years after surgery [42, 45–48]

Although some studies found no influence on sexual function after surgery, most studies did. IRA was generally associated with better sexual function compared to IPAA. On the other hand, regardless of having an IRA or IPAA, women reported impaired sexual desire and enjoyment, dysfunctional lubrication, and difficulty to orgasm, while men reported impotence or retrograde ejaculation. Both men and women reported pain during intercourse. Moreover, negative influences on perceived physical attractiveness, feelings of femininity or masculinity, and dissatisfaction with the body were also reported [18, 26, 49–52]

### Life with a polyposis syndrome

Awareness of body appearance and surgery-related symptoms was described. Sex was difficult post-surgery due to the influence on body image and bowel movements, and intimate relationships were avoided. Poorer general health was described, and scoring differed between females and males. Body image seemed not to be associated with HRQoL, but health and functioning scales were scored lower compared to the general population. The referred articles in this paragraph include both individuals having gone through surgery, not having gone through surgery, or status being unclear [27, 29–31, 33]

### General well-being from the patients’ perspectives

#### Genetic investigation

Experiences of both work and social discrimination were described. Concerns regarding work-related issues included missing job opportunities, pay increases that were related to hospital visits, sickness benefits, and insurance issues due to their diagnosis. There was also a concern about regularly being away from work and perceiving negative attitudes from colleagues and managers. Patients also described a sense of social discrimination, i.e., people around them lacked understanding of what it is like to live with a polyposis syndrome. The desire to have children was in many ways affected by their diagnosis, i.e., they wanted fewer children or none at all, partly because the disease is hereditary but also due to the risk of becoming ill in the future [18, 54].

#### Life after surgery

Overall, patients with FAP undergoing surgery perceived themselves as being in good health, optimistic about life the future, and they felt highly self-efficacious. Social support was perceived as even better compared with population norms. The support by families and friends helped patients come to terms with their disease and be optimistic about their ability to cope with the upcoming challenges post-surgery. On the other hand, social activities, education, friendships, relationships with other family members, as well as intimate relationships and the willingness to have children were also described to be negatively influenced. Altered physical health could lead to an interference with leisure time activities and a need for an altered lifestyle, including dietary restrictions and work life. Examples of dietary restrictions included modifications in eating habits to avoid bowel movements when joining social activities. Work-life balance seemed affected by surgery, as some patients never returned to work, returned a year after surgery, had to change jobs, or took a part-time job. This seemed to be the case especially for patients undergoing surgery at an older age or receiving a second IPAA.

In comparing general health in patients with IRA, IPAA, and ileostomy, IRA was reported to be favourable regarding sports, social activities, recreation, work around the house, family relationships, and travel compared to IPAA. Strong coping efforts were required for those with IPAA to come to terms with the resulting impairments in everyday life after ileostomy closure. One study found improved general health in patients with an ileostomy compared to those with IRA and IPAA despite having to adhere to food restrictions to a higher extent. An additional surgery, such as duodenectomy, in patients with IPAA or IPAA had no influence on life, except for those with new-onset diabetes post-surgery [24, 26, 39, 41–45, 49, 51–53]

#### Life with a polyposis syndrome

It was described that families were brought closer together and that FAP led to more openness, but at the same time a lack of understanding was mentioned. A desire for family support was highlighted. Concerns for family members and future children were evident. One study showed that family functioning was high and that marital status predicted HRQoL. Employment was impacted and specifically discussed among younger individuals. Social activities were affected and sometimes avoided. Being female and having negative beliefs related to the disease contributed to risks for limitations in daily life. A strong belief in marriage was found, but at the same time a lack of understanding from partners or ended relationships due to diagnosis and surgery was described. Fertility concerns were evident, and both a desire to have children and a desire not to have children were described. Females were less often parents, termination of pregnancy was described as acceptable, and it was believed that family and social support could reduce fertility-related distress [29–33, 36]

## Discussion

### Summary of evidence

This scoping review aimed to synthesise the evidence on mental and physical health, and well-being in individuals with polyposis syndromes. A total of 39 studies were included. The synthesis revealed three main topics: the period of genetic investigation, the life after surgery, and the life with a polyposis syndrome. The topics were further analysed regarding the patient’s perspective on mental and physical health and well-being, according to the aim. For the interpretation of the results, it is notable that of the 39 studies, only four included participants with PJS, and the rest included FAP patients. There are other polyposis syndromes such as MUTYH-associated polyposis (MAP) or Juvenile Polyposis (JP), but none of the selected studies involved patients with these conditions. The findings show that patients exhibited mental and physical health in parity with population norms when using generic quality of life questionnaires. However, feelings of vulnerability, anxiety, worry and fear, especially in younger participants, were reported, as well as decreased sexual function, bowel function, food restrictions, dissatisfaction with the body, and physical attractiveness. Regarding well-being, an altered lifestyle, work and social discrimination, insurance matters, concerns for family members, relationship issues, and fertility concerns were evident. Being female seemed to be related to mental health issues or vulnerability thereof.

#### Mental health from the patient’s perspective

In general, patients with polyposis syndromes exhibited comparable mental health to population norms, however, anxiety and feelings of uncertainty were related to the period of genetic testing; feelings of intrusion, worry about cancer, and fear of complications were related to surgery and feelings of isolation; and embarrassment and guilt were related to life with a polyposis syndrome. A recent review found that there was some evidence for increased distress and anxiety during the time for genetic testing and surgery among patients with FAP [55]. Moreover, Karstensen and colleagues [56] compared patients with FAP to matched controls and found an increased risk of developing mood and behavioural disorders, with an increased likelihood of needing antidepressants and antipsychotics when having FAP. In the current study, several factors were found to be important for how patients perceived their mental health, such as low levels of optimism and self-esteem in children, young age regarding cancer worry, and older age when it comes to bowel function after surgery. This is somewhat also supported by the results of Mol and colleagues [55], who reported factors such as negative beliefs, risk perception, social status (including low education), being single, and low family function as important predictors for mental health. Maladaptive family function and lack of support from friends have previously been reported to increase distress during the period of genetic testing in other patients with hereditary cancer syndromes [57].

#### Physical health from the patient’s perspective

Physical health was influenced by surgery and, predominantly, the life after surgery in patients with FAP. The challenge is associated with the fact that patients consider themselves healthy with the risk of complications and symptoms post-surgery after removing healthy organs [58]. When measuring physical health with a generic questionnaire such as the SF-36, physical health was comparable with population norms. However, when using surgery-specific questionnaires, influence on sexual function, decreased bowel function, food restrictions, dissatisfaction with the body, and physical attractiveness were reported. Factors such as type of and a second surgery, older age, as well as perceived disability and female gender were reported to be important for perceived physical health. Interestingly, negative impact on body image was not only reported by patients with FAP that had had surgery but also by those that had not.

#### General well-beingfrom the patient’s perspective

In terms of well-being, patients described issues relating to an altered lifestyle, work and social discrimination, insurance matters, interference with leisure time activities, concerns for family members, ended relationships, and changed desires about wanting children. Factors such as the potential risk of having cancer, risk-reducing surgery, and worry among young patients may disturb life plans. This has previously been reported in adolescents and young adults with cancer [59], and findings by Karstensen and colleagues [56] showed that patients with FAP had significantly lower levels of education compared to matched controls without FAP. It is, however, worth noting that some patients also perceived social support as even better compared with population norms.

### Study limitations and strengths

A strength of the present study is that the PRISMA-ScR checklist to ensure transparent reporting [11] was used. Moreover, a scoping review is suitable when the focus is to map the evidence rather than to assess it. No quality assessments of included articles were performed, however, the results are based on articles considered to be of relevant quality (impact factor 1.4–12.5, 82% > 2). The search strategy was constructed in collaboration with librarians at a medical university library with a vast knowledge of how to conduct systematic literature searches. Moreover, the few authors involved in the reviewing process reduced the risk of bias. The authors contributed with diverse expertise in certain areas, such as nursing, medicine, and genetic counseling, when reviewing the articles. Authors (AJ and KF) have experience working with directed content analysis, as described by Hsieh and Shannon [15], which is why aspects of that analytical approach were used in the present work. Co-authors (VH and SWB) were introduced and briefed about the directed content analysis beforehand, and findings were discussed among authors until consensus was reached. Some findings tend to overlap between the subgroups chosen by the authors (genetic testing, surgery, and life), but special effort was taken to present the findings as exclusively as possible.

This study reports findings mostly from individuals with FAP (95%). Only a few studies include participants with Peutz Jehgers syndrome and no studies were found relevant to this scoping review that included participants with MAP or Juvenile polyposis. This is in part explained by the fact that FAP is more common than the other polyposis syndrome. The decision to include studies on PJS is based on the fact that, despite the differences, there are similarities regarding the hereditary nature of the illnesses and extensive examination programs. The four studies on PJS included in the study concerned QoL and reproductive issues relevant for patients with hereditary polyposis syndromes. From a caregiver’s perspective, the time of genetic testing and the time of surgery might be seen as the two most pivotal aspects in the life of having a polyposis syndrome, which is probably why most studies conducted have focused on these two topics. The included studies amount to less than 1% of all articles generated by the literature search. This is, however, a result on par with scoping reviews on similar topics [60, 61]. The vast majority of studies had a quantitative design and focused mainly on surgical aspects. Most studies were published before 2013, which could be reflected by the fact that surgical techniques and principles for this group of patients have not changed drastically in the last decade. The findings from this scoping review are based only on studies published in English and mainly on studies performed in high-income countries, so there is a possibility that important studies in other languages are missed and that certain ethnic patient populations may be excluded from this review.

### Clinical implications

The current study found several areas of concern and factors important for mental and physical health and well-being in patients with polyposis syndromes, especially FAP. To capture this in clinical settings, appropriate screening tools, such as relevant patient-reported outcome measures (PROM), should be developed and implemented. Therefore, our research group will continue the work to develop such a tool. The results of PROM could guide person-centered care and the organization of health care to meet patients needs. Moreover, the present review can act as a foundation for further studies. Special attention is needed for the more infrequent polyposis syndromes, represented to a limited extent in the current study. In addition, most studies found to be relevant for this review were published up to 2013, while newer studies are needed in order to build to the body of knowledge regarding mental and physical health and well-being. This review could also be adapted into educational efforts for healthcare professionals. Since 2013, social media has gained a large platform in terms of healthcare services and patient support groups. This represents a unique opportunity both for patients who may not have been able to share experiences before and also for healthcare professionals encountering these patients and their significant others. Digital aids may support both healthcare professionals and patients, especially those living in rural areas with long travel distances. We have already developed such a support tool for individuals invited to colorectal cancer screening, with information presented in various ways and adjusted to individuals living with different functional limitations [62, 63]. Based on previous experiences and the present findings, we believe that we can design a relevant and informative tool for this group of patients, applying a co-creation approach.

## Conclusion

Patients with polyposis syndromes exhibited health as population norms. The current study shows that the reality of patients lives is more complex, and this may not be fully captured by measuring quality of life with generic measurement tools. Being young increased cancer worry, influenced physical health negatively, older age was related to decreased bowel function after surgery, and decreased sexual function was reported in both men and women after cancer risk-reducing surgery. Concerns for family members were described, as well as interference with work and social life. In addition, being female seemed to be related to mental health issues or vulnerability thereof To capture outcomes important for patients with polyposis syndromes, newer studies are needed to develop tools, such as patient-reported outcome measures (PROM). In addition, studies to develop and implement digital support tools are also of importance since health care providers often lack knowledge in polyposis syndromes.

## Supplementary Information

Below is the link to the electronic supplementary material.

Supplementary Material 1

Supplementary Material 2

## Data Availability

The data are available upon request to the authors.
