# Supplementary material for: Mental- and physical health, and general well-being in patients with polyposis syndromes: a scoping review
Source: Fam Cancer. 2026 Feb 14;25(1):20. doi: 10.1007/s10689-026-00537-4 (PMC12906515; doi:10.1007/s10689-026-00537-4)
Supplement: Supplementary file 1 — Supplementary Material 1 [file 10689_2026_537_MOESM1_ESM.docx]

1. Medline

| Interface: Ovid MEDLINE(R) ALL  Date of Search: 2024-10-22  Number of hits: 1764  Comment: In Ovid, two or more words are automatically searched as phrases; i.e. no quotation marks are needed | Field labels   - exp/ = exploded MeSH term - / = non exploded MeSH term - .ti,ab,kf. = title, abstract and author keywords - adjx = within x words, regardless of order - * = truncation of word for alternate endings |
| --- | --- |
| Ovid MEDLINE(R) ALL <1946 to October 21, 2024>   \| 1 \| exp Adenomatous Polyposis Coli/ \| 6931 \| \| --- \| --- \| --- \| \| 2 \| Genes, APC/ \| 2708 \| \| 3 \| Smad4 Protein/ \| 2716 \| \| 4 \| Adenomatous Polyposis Coli Protein/ \| 2312 \| \| 5 \| (Polypos* adj3 (familial or myh* or Adenomato* or juvenile or hereditary)).ti,ab,kf. \| 11503 \| \| 6 \| (Adenomato* adj3 (coli or colon or colorectal)).ti,ab,kf. \| 4748 \| \| 7 \| gardner* syndrome*.ti,ab,kf. \| 1149 \| \| 8 \| Peutz-Jeghers Syndrome/ \| 2072 \| \| 9 \| ((Peutz adj2 Jegher*) or Perior* Lentiginosis or "Polyps and Spots" or pigmented spot or APC gene*).ti,ab,kf. \| 4886 \| \| 10 \| Juvenile polyposis syndrome.rs. \| 157 \| \| 11 \| (Smad4 or smad 4 or dpc4 or "sma and mad*" or LKB1 or LKB 1 or STK11 or STK 11 or serine threonine kinase 11 or BMPR1A).ti,ab,kf. \| 10378 \| \| 12 \| or/1-11 \| 29416 \| \| 13 \| Adenomatous Polyps/ \| 2085 \| \| 14 \| Intestinal Polyposis/ \| 737 \| \| 15 \| exp Intestinal Polyps/ \| 16890 \| \| 16 \| ((adenomatous or intestinal or colonic) adj1 (polyps or polypos*)).ti,ab,kf. \| 13708 \| \| 17 \| or/13-16 \| 28818 \| \| 18 \| Heredity/ \| 5027 \| \| 19 \| (Heredit* or familial).ti,ab,kf. \| 220046 \| \| 20 \| or/18-19 \| 221505 \| \| 21 \| 17 and 20 \| 6808 \| \| 22 \| 21 or 12 \| 29685 \| \| 23 \| Patient Reported Outcome Measures/ \| 16491 \| \| 24 \| exp Health Status/ \| 497564 \| \| 25 \| exp Attitude to Health/ \| 486421 \| \| 26 \| exp Health Surveys/ \| 640341 \| \| 27 \| Health Literacy/ \| 10556 \| \| 28 \| Self Report/ \| 46545 \| \| 29 \| Qualitative Research/ \| 93990 \| \| 30 \| Interviews as Topic/ \| 70042 \| \| 31 \| psychology.fs. \| 1238439 \| \| 32 \| ((patient or self) adj2 (rated or rating* or report* or percept* or perceiv* or satisfaction or scale* or survey* or questionnaire* or experienc*)).ti,ab,kf. \| 536847 \| \| 33 \| (PRO or PROs or PROM or PROMs).ti,ab,kf. \| 298962 \| \| 34 \| ((disease or health* or illness* or symptom*) adj3 (belief* or literacy or measure* or questionnaire* or scale* or status)).ti,ab,kf. \| 350914 \| \| 35 \| (quality of life or qol or wellbeing or well being).ti,ab,kf. \| 570383 \| \| 36 \| (psychosocial* or anxiet*).ti,ab,kf. \| 412128 \| \| 37 \| (risk perception* or risk awareness*).ti,ab,kf. \| 11496 \| \| 38 \| qualitative.ti,ab,kf. \| 369367 \| \| 39 \| exp Family Relations/ \| 105761 \| \| 40 \| ((Family or ((parent or mother or father) adj2 (child* or offspring*)) or Intergeneration*) adj2 (Relation* or dynamic* or communicat*)).ti,ab,kf. \| 27972 \| \| 41 \| interview*.ti,ab,kf. \| 497846 \| \| 42 \| exp Cost of Illness/ \| 35477 \| \| 43 \| ((cost* or econom*) adj3 (burden or illness* or disease* or sickness)).ti,ab,kf. \| 39747 \| \| 44 \| or/23-43 \| 4026069 \| \| 45 \| 22 and 44 \| 1764 \| | |

2. Embase

| Interface: embase.com  Date of Search: 2024-10-22  Number of hits: 2739  Comment: Emtree is the controlled vocabulary in Embase | Field labels   - /exp = exploded Emtree term - /de = non exploded Emtree term - ti,ab,kw = title, abstract and author keywords - NEAR/x = within x words, regardless of order - * = truncation of word for alternate endings |
| --- | --- |
| \| No. \| Query \| Results \| \| --- \| --- \| --- \| \| #45 \| #44 AND #43 \| 2739 \| \| #44 \| #11 OR #20 \| 54894 \| \| #43 \| #21 OR #22 OR #23 OR #24 OR #25 OR #26 OR #27 OR #28 OR #29 OR #30 OR #31 OR #32 OR #33 OR #34 OR #35 OR #36 OR #37 OR #38 OR #39 OR #40 OR #41 OR #42 \| 3749946 \| \| #42 \| ((parent OR mother OR father) NEAR/2 (child* OR offspring*) NEAR/2 (relation* OR dynamic* OR communicat*)):ti,ab,kw \| 8441 \| \| #41 \| ((family OR intergeneration*) NEAR/2 (relation* OR dynamic* OR communicat*)):ti,ab,kw \| 23112 \| \| #40 \| ((cost* OR econom*) NEAR/3 (burden OR illness* OR disease* OR sickness)):ti,ab,kw \| 60482 \| \| #39 \| 'cost of illness'/de \| 21854 \| \| #38 \| 'family relation'/exp \| 137112 \| \| #37 \| 'quality of life'/exp \| 719645 \| \| #36 \| 'interview':ti,ab,kw \| 239560 \| \| #35 \| qualitative:ti,ab,kw \| 451253 \| \| #34 \| 'risk perception*':ti,ab,kw OR 'risk awareness*':ti,ab,kw \| 13357 \| \| #33 \| psychosocial*:ti,ab,kw \| 185790 \| \| #32 \| 'quality of life':ti,ab,kw OR qol:ti,ab,kw \| 651255 \| \| #31 \| ((disease OR health* OR illness* OR symptom*) NEAR/3 (belief* OR literacy OR measure* OR questionnaire* OR scale* OR status)):ti,ab,kw \| 493846 \| \| #30 \| pro:ti,ab,kw OR pros:ti,ab,kw OR prom:ti,ab,kw OR proms:ti,ab,kw \| 450536 \| \| #29 \| ((patient OR self) NEAR/2 (rated OR rating* OR report* OR percept* OR perceiv* OR satisfaction OR scale* OR survey* OR questionnaire* OR experienc*)):ti,ab,kw \| 757796 \| \| #28 \| 'interview'/exp \| 405516 \| \| #27 \| 'qualitative research'/exp \| 135976 \| \| #26 \| 'self report'/de \| 166604 \| \| #25 \| 'health literacy'/exp \| 23468 \| \| #24 \| 'health survey'/exp \| 295132 \| \| #23 \| 'attitude to health'/de \| 139148 \| \| #22 \| 'health status'/exp \| 345863 \| \| #21 \| 'patient-reported outcome'/exp \| 67829 \| \| #20 \| #16 AND #19 \| 19684 \| \| #19 \| #17 OR #18 \| 4285841 \| \| #18 \| heredit*:ti,ab,kw OR familial:ti,ab,kw \| 305150 \| \| #17 \| 'heredity'/exp \| 4144478 \| \| #16 \| #12 OR #13 OR #14 OR #15 \| 56870 \| \| #15 \| ((adenomatous OR intestinal OR colonic) NEAR/1 (polyps OR polypos*)):ti,ab,kw \| 20334 \| \| #14 \| 'intestine polyp'/exp \| 45517 \| \| #13 \| 'intestinal polyposis'/exp \| 18963 \| \| #12 \| 'adenomatous polyp'/de \| 10755 \| \| #11 \| #1 OR #2 OR #3 OR #4 OR #5 OR #6 OR #7 OR #8 OR #9 OR #10 \| 50804 \| \| #10 \| smad4:ti,ab,kw OR 'smad 4':ti,ab,kw OR dpc4:ti,ab,kw OR 'sma and mad*':ti,ab,kw OR lkb1:ti,ab,kw OR 'lkb 1':ti,ab,kw OR stk11:ti,ab,kw OR 'stk 11':ti,ab,kw OR 'serine threonine kinase 11':ti,ab,kw OR bmpr1a:ti,ab,kw \| 16692 \| \| #9 \| 'juvenile polyposis'/de \| 342 \| \| #8 \| ((peutz NEAR/2 jegher*):ti,ab,kw) OR 'perior* lentiginosis':ti,ab,kw OR 'polyps and spots':ti,ab,kw OR 'pigmented spot':ti,ab,kw OR 'apc gene*':ti,ab,kw \| 6796 \| \| #7 \| 'peutz-jeghers syndrome'/de \| 4008 \| \| #6 \| 'gardner* syndrome*':ti,ab,kw \| 533 \| \| #5 \| (adenomato* NEAR/3 (coli OR colon OR colorectal)):ti,ab,kw \| 6107 \| \| #4 \| (polypos* NEAR/3 (familial OR myh* OR adenomato* OR juvenile OR hereditary)):ti,ab,kw \| 15647 \| \| #3 \| 'apc protein'/de \| 8564 \| \| #2 \| 'smad4 protein'/de \| 9394 \| \| #1 \| 'colon polyposis'/exp \| 13304 \| | |

3. Web of Science Core Collection

| Interface: Clarivate Analytics  Editions = A&HCI , ESCI , SCI-EXPANDED , SSCI  Date of Search: 2024-10-22  Number of hits: 1158 | Field labels   - TS/Topic = title, abstract, author keywords and Keywords Plus - NEAR/x = within x words, regardless of order - * = truncation of word for alternate endings   Note: the *Exact search*-function was used for all the searches |
| --- | --- |
| \| # \| Search Query \| Results \| \| --- \| --- \| --- \| \| 1 \| TS=(Adenomato* NEAR/3 (coli OR colon OR colorectal )) \| 5834 \| \| 2 \| TS="gardner* syndrome*" \| 948 \| \| 3 \| TS=(Smad4 OR "smad 4" OR dpc4 OR "sma and mad*" OR LKB1 OR "LKB 1" OR STK11 OR "STK 11" OR "serine threonine kinase 11" OR BMPR1A ) \| 12082 \| \| 4 \| #1 OR #2 OR #3 \| 18668 \| \| 5 \| TS=((adenomatous OR intestinal OR colonic ) NEAR/1 (polyps OR polypos* )) \| 18847 \| \| 6 \| TS=(Heredit* or familial) \| 271381 \| \| 7 \| #5 AND #6 \| 8776 \| \| 8 \| #4 OR #7 \| 25382 \| \| 9 \| TS=((patient OR self) NEAR/2 (rated OR rating* OR report* OR percept* OR perceiv* OR satisfaction OR scale* OR survey* OR questionnaire* OR experienc*)) \| 676119 \| \| 10 \| TS=(PRO OR PROs OR PROM OR PROMs) \| 396967 \| \| 11 \| TS=((disease OR health* OR illness* OR symptom*) NEAR/3 (belief* OR literacy OR measure* OR questionnaire* OR scale* OR status)) \| 433354 \| \| 12 \| TS=("quality of life" OR qol OR psychosocial* OR "risk perception*" OR "risk awareness*" OR qualitative OR interview*) \| 1897776 \| \| 13 \| TS=((Family OR ((parent OR mother OR father ) NEAR/2 (child* OR offspring* )) OR Intergeneration* ) NEAR/2 (Relation* OR dynamic* OR communicat* )) \| 52337 \| \| 14 \| TS=((cost* OR econom* ) NEAR/3 (burden OR illness* OR disease* OR sickness )) \| 56216 \| \| 15 \| #9 OR #10 OR #11 OR #12 OR #13 OR #14 \| 3119060 \| \| 16 \| #15 AND #8 \| 1158 \| | |

4. Cinahl

| Interface: Ebsco  Date of Search: 2024-10-22  Number of hits: 610 | Field labels   - MH+ = exploded Cinahl Heading - MH = non exploded Cinahl Heading - TI = title - AB = abstract - Nx = within x words, regardless of order - * = truncation of word for alternate endings |
| --- | --- |
| \| # \| Query \| Results \| \| --- \| --- \| --- \| \| S46 \| S44 AND S45 \| 610 \| \| S45 \| S9 OR S18 \| 3,813 \| \| S44 \| S19 OR S20 OR S21 OR S22 OR S23 OR S24 OR S25 OR S26 OR S27 OR S28 OR S29 OR S30 OR S31 OR S32 OR S33 OR S34 OR S35 OR S36 OR S37 OR S38 OR S39 OR S40 OR S41 OR S42 OR S43 \| 1,882,959 \| \| S43 \| (((TI cost* OR AB cost*) OR (TI econom* OR AB econom*)) N3 ((TI burden OR AB burden) OR (TI illness* OR AB illness*) OR (TI disease* OR AB disease*) OR (TI sickness OR AB sickness))) \| 13,838 \| \| S42 \| (MH "Economic Aspects of Illness") \| 11,117 \| \| S41 \| (TI interview* OR AB interview*) \| 269,737 \| \| S40 \| ((((TI parent OR AB parent) OR (TI mother OR AB mother) OR (TI father OR AB father)) N2 ((TI child* OR AB child*) OR (TI offspring* OR AB offspring*))) N2 ((TI Relation* OR AB Relation*) OR (TI dynamic* OR AB dynamic*) OR (TI communicat* OR AB communicat*))) \| 4,981 \| \| S39 \| (((TI Family OR AB Family) OR (TI Intergeneration* OR AB Intergeneration*)) N2 ((TI Relation* OR AB Relation*) OR (TI dynamic* OR AB dynamic*) OR (TI communicat* OR AB communicat*))) \| 14,925 \| \| S38 \| (MH "Family Relations+") \| 85,400 \| \| S37 \| (TI qualitative OR AB qualitative) \| 185,944 \| \| S36 \| ((TI "risk perception*" OR AB "risk perception*") OR (TI "risk awareness*" OR AB "risk awareness*")) \| 4,405 \| \| S35 \| ((TI psychosocial* OR AB psychosocial*) OR (TI anxiet* OR AB anxiet*)) \| 167,026 \| \| S34 \| ((TI "quality of life" OR AB "quality of life") OR (TI qol OR AB qol) OR (TI wellbeing OR AB wellbeing) OR (TI "well being" OR AB "well being")) \| 235,979 \| \| S33 \| (((TI disease OR AB disease) OR (TI health* OR AB health*) OR (TI illness* OR AB illness*) OR (TI symptom* OR AB symptom*)) N3 ((TI belief* OR AB belief*) OR (TI literacy OR AB literacy) OR (TI measure* OR AB measure*) OR (TI questionnaire* OR AB questionnaire*) OR (TI scale* OR AB scale*) OR (TI status OR AB status))) \| 158,275 \| \| S32 \| ((TI PRO OR AB PRO) OR (TI PROs OR AB PROs) OR (TI PROM OR AB PROM) OR (TI PROMs OR AB PROMs)) \| 38,770 \| \| S31 \| (((TI patient OR AB patient) OR (TI self OR AB self)) N2 ((TI rated OR AB rated) OR (TI rating* OR AB rating*) OR (TI report* OR AB report*) OR (TI percept* OR AB percept*) OR (TI perceiv* OR AB perceiv*) OR (TI satisfaction OR AB satisfaction) OR (TI scale* OR AB scale*) OR (TI survey* OR AB survey*) OR (TI questionnaire* OR AB questionnaire*) OR (TI experienc* OR AB experienc*))) \| 347,575 \| \| S30 \| MW "pf" \| 570,560 \| \| S29 \| (MH "Interviews+") \| 267,095 \| \| S28 \| (MH "Qualitative Studies+") \| 196,69 \| \| S27 \| (MH "Self Report+") \| 90,935 \| \| S26 \| (MH "Health Literacy") \| 8,172 \| \| S25 \| (MH "Surveys+") \| 275,799 \| \| S24 \| (MH "Survey Research") \| 20,898 \| \| S23 \| (MH "Attitude to Illness+") \| 28,466 \| \| S22 \| (MH "Attitude to Health+") \| 187,073 \| \| S21 \| (MH "Health Status+") \| 146,160 \| \| S20 \| (MH "Outcome Assessment") \| 53,908 \| \| S19 \| (MH "Patient-Reported Outcomes") \| 8,528 \| \| S18 \| S16 AND S17 \| 738 \| \| S17 \| S14 OR S15 \| 55,134 \| \| S16 \| S10 OR S11 OR S12 OR S13 \| 4,535 \| \| S15 \| ((TI Heredit* OR AB Heredit*) OR (TI familial OR AB familial)) \| 31,532 \| \| S14 \| (MH "Genetics") \| 24,994 \| \| S13 \| (((TI adenomatous OR AB adenomatous) OR (TI intestinal OR AB intestinal) OR (TI colonic OR AB colonic)) N1 ((TI polyps OR AB polyps) OR (TI polypos* OR AB polypos*))) \| 1,581 \| \| S12 \| (MH ”Intestinal Polyps+”) \| 3,075 \| \| S11 \| (MH ”Intestinal Polyposis”) \| 185 \| \| S10 \| (MH ”Adenomatous Polyps”) \| 369 \| \| S9 \| S1 OR S2 OR S3 OR S4 OR S5 OR S6 OR S7 OR S8 \| 3,775 \| \| S8 \| ((TI Smad4 OR AB Smad4) OR (TI "smad 4" OR AB "smad 4") OR (TI dpc4 OR AB dpc4) OR (TI "sma and mad*" OR AB "sma and mad*") OR (TI LKB1 OR AB LKB1) OR (TI "LKB 1" OR AB "LKB 1") OR (TI STK11 OR AB STK11) OR (TI "STK 11" OR AB "STK 11") OR (TI "serine threonine kinase 11" OR AB "serine threonine kinase 11") OR (TI BMPR1A OR AB BMPR1A)) \| 869 \| \| S7 \| (((TI Peutz OR AB Peutz) N2 (TI Jegher* OR AB Jegher*)) OR (TI "Perior* Lentiginosis" OR AB "Perior* Lentiginosis") OR (TI "Polyps and Spots" OR AB "Polyps and Spots") OR (TI "pigmented spot" OR AB "pigmented spot") OR (TI "APC gene*" OR AB "APC gene*")) \| 432 \| \| S6 \| (MH ”Peutz-Jeghers Syndrome”) \| 252 \| \| S5 \| (TI "gardner* syndrome*" OR AB "gardner* syndrome*") \| 91 \| \| S4 \| ((TI Adenomato* OR AB Adenomato*) N3 ((TI coli OR AB coli) OR (TI colon OR AB colon) OR (TI colorectal OR AB colorectal))) \| 407 \| \| S3 \| ((TI Polypos* OR AB Polypos*) N3 ((TI familial OR AB familial) OR (TI myh* OR AB myh*) OR (TI Adenomato* OR AB Adenomato*) OR (TI juvenile OR AB juvenile) OR (TI hereditary OR AB hereditary))) \| 1,002 \| \| S2 \| (MH ”Genes, Tumor Suppressor”) \| 1,080 \| \| S1 \| (MH ” Adenomatous Polyposis Coli+”) \| 862 \| | |
