# Supplementary material for: Mental- and physical health, and general well-being in patients with polyposis syndromes: a scoping review
Source: Fam Cancer. 2026 Feb 14;25(1):20. doi: 10.1007/s10689-026-00537-4 (PMC12906515; doi:10.1007/s10689-026-00537-4)
Supplement: Supplementary file 2 — Supplementary Material 2 [file 10689_2026_537_MOESM2_ESM.docx]

| **[Reference], first author, date** | **Country** | **Aim** | **Design** | **Analyzed, n** | **Syndrome** |
| --- | --- | --- | --- | --- | --- |
| [1] Alhassan, 2024 | Saudi Arabia | To explore surveillance compliance in our population, as well as the disease impact on their quality of life (QoL). | cross sectional,  telephone interviews: questionnaires SF-36, EORTC | 14 | FAP |
| [2] Andrews, 2006 | Australia | This study explores the attitudes of young adults affected by or at risk of developing FAP regarding the current protocols of genetic testing and attitudes toward reproductive technologies and genetic testing of unborn offspring and assesses the information and support needs among this group. The prevalence and nature of discrimination as a result of FAP will also be investigated. | cross sectional,  study specific questionnaire assessing attitudes toward genetic testing, information sources and support needs | 88 | FAP |
| [3] Andrews, 2007 | Australia | The current study was designed to assess the impact of being diagnosed with FAP or being at one-in-two risk, on a number of life domains, and to explore the impact of different surgical procedures and sociodemographic variables on psychologic adjustment. | cross sectional,  study specific questionnaires measuring quality of life and life impact; The Impact of Event Scale | 88 | FAP |
| [4] Christou, 2016 | UK | The aim of the study was to retrospectively assess the quality of life in these patients, to evaluate the long-term functional outcome of the procedure and to compare the results with respect to early and late postoperative complications between UC and FAP patients. The secondary endpoint is to identify possible factors associated with good and less satisfactory functional results. | retrospective, 1987-2006,    questionnaires: structured questionnaire regarding complications, SF-36, Wexner score | 56 | FAP |
| [5] Codori, 2003 | US | In the present study, we evaluated the long-term psychological effects of genetic testing for FAP in the previously studied cohort of children and parents, up to 4 years after testing. | longitudinal; 3, 12, 23-55 months after disclosure test,  the Children’s Depression Inventory, Reynold’s Adolescent Depression Scale (RADS; | 22 | FAP |
| [6] Collard, 2020 | France | The aim was to assess the long-term consequences of pancreaticoduodenectomy on bowel function, as well as the consequences on the QoL of FAP patients with an IPAA. | retrospective, 1991 and 2017,  questionnaires:  The EORTC-QLQ-30, The Wexner score | 32 PD+IPAA/32 IPAA | FAP |
| [7] Danieli, 2024 | Canada | This study investigated the neurodevelopmental impact of pathogenic adenomatous polyposis coli (APC) gene variants in patients with familial adenomatous polyposis (FAP), a cancer predisposition syndrome. | cross sectional,  The Social Responsiveness Scale (SRS), | 66 FAP/34 siblings | FAP |
| [8] van Duijvendijk, 2000 | The Netherlands | To assess the QoL with validated measures in a large series of patients with FAP having either an ileorectal anastomosis or a proctocolectomy and ileal pouch-anal anastomosis. | retrospective, 1961-1996,  questionnaires: SF-36, EORTC QLQ-CR38, | 118 (IPAA)/161 (IRA) | FAP |
| [9] Douma, 2010 | The Netherlands | The primary objective was to establish the prevalence of heightened levels of generalized and situational distress among individuals belonging to FAP families, to identify sociodemographic, clinical and psychosocial factors significantly associated with situational distress, and the perceived need for and use of professional psychosocial support was investigated. | cross sectional,  questionnaire: Mental Health Index-5;  study specific questionnaire measuring three levels of psychological distress were assessed: (1) generalized; (2) cancer-specific; and (3) FAP-specific. | 341 | FAP |
| [10] Douma, 2010 | The Netherlands | To report on attitudes toward childhood DNA testing, PND and PGD among carriers, individuals at risk, and noncarriers from FAP families | cross sectional,  study specific questionnaires measuring attitudes toward and experiences of DNA testing, attitudes toward and experience of PND and PGD, psychosocial variables | 65 | FAP |
| [11] Durno, 2012 | Canada | The aim of the current study was to investigate the functional outcomes and quality of life in patients with FAP who had undergone surgery at a very young age. | cross sectional,  questionnaires: The Impact of Event Scale, SF-12, study specific Cancer-Related Worry scale, the Rosenberg Self-Esteem Scale | 28 (21/7) | FAP |
| [12] Eriksson, 2016 | Sweden | The aim of the study was to examine patients’ beliefs about having familial adenomatous polyposis (FAP), a hereditary colorectal cancer syndrome, and how these beliefs are associated with adherence to endoscopic surveillance. | cross sectional,  questionnaire:  The Illness Perception Questionnaire | 209 | FAP |
| [13] Erkek, 2007 | US | The aim of the present study was to elucidate the influence of a younger age at the time of surgery on the functional outcome and quality of life in patients undergoing IPAA for FAP. | longitudinal; 1, 3, 5 years post surgery,  questionnaires: Cleveland GlobalQuality of Life (CGQL) | 53 | FAP |
| [14] Esplen, 2004 | Canada | The purpose of this study was to examine the health-related quality of life in a sample of Canadian adults diagnosed with FAP and desmoid tumors. | cross sectional,  focus group discussion  questionnaire: The Body Esteem Measure, The Social Support Questionnaire (SSQ), McMaster Family Assessment Device (FAD), Beck Hopelessness Scale (BHS), Quality of Life Index (QLI) | 30 | FAP |
| [15] Fritzell, 2010 | Sweden | The aim of the present study was to gain a deeper understanding of how FAP affects life by exploring patients’ views of what it is like living with the condition and having to be committed to a lifelong screening program. | cross sectional,  focus group discussions | 14 | FAP |
| [16] Fritzell, 2011 | Sweden | The aim was to investigate self-reporting by adults with FAP with regard to presence, frequency, and troublesomeness of abdominal symptoms in relation to health status. An additional aim was to study abdominal symptoms in relation to gender and type of colorectal surgery performed. | cross sectional,  questionnaires: the Abdominal Symptom Questionnaire, SF-36 | 209 | FAP |
| [17] Ganschow, 2010 | Germany | The purpose of the present study was to analyze the long-term results 10 and more years after IPAA for patients with FAP, exclusively. To compare QoL of postoperative patients with that of the healthy German population and to monitor the impact of specific disease- and condition-related factors, | long term >10 years,  questionnaires:  SF-36, the disease-specific Gastrointestinal Quality of Life Index, 10 items of disease-specific factors | 80 | FAP |
| [18] Ganschow, 2018 | Germany | To analyze perioperative and long-term outcome after PD and PPTD for FAP-associated duodenal adenomatosis, including QoL and recurrence of adenomas in the neoduodenum after PPTD. | prospective, 1992-2012,  SF-36 | 38 | FAP |
| [19] Gunther, 2002 | Germany | Against this background, the present study is a direct comparison of IRA and IPAA in terms of quality of life with emphasis on bowel function in FAP patients. | cross sectional,  questionnaires: Jorge-Wexner, Jostarndt (JS)-scores, The functional score of Öresland, Pemberton (PS) | 59 | FAP |
| [20] Hassan, 2005 | US | The purpose of this study was to use such standardized instruments to examine the complications, functional results, and HRQL of FAP patients who had an IPAA or an IRA during the same period at our institution. | retrospective, 1981-1998,  questionnaires: study-specific quality-of-life questionnaire, SF-36 | 115 | FAP |
| [21] Ko, 2001 | US | The purpose of this study is to evaluate FAP patients who underwent prophylactic (procto)colectomy with either permanent ileostomy or restored bowel continuity reconstruction. The functional outcomes of both groups are reported, and the formal HRQL assessments are compared. | cross sectional,  questionnaire:  SF-36 | 54 | FAP |
| [22] Ko, 2000 | US | The purpose of this study was to determine whether any relationship exists between functional result and HRQL in patients with FAP after IRA and IPAA. | retrospective cohort, 1980-1998  questionnaire:  SF-36 | 44 | FAP, |
| [23] Krausz, 2005 | Israel | To evaluate the early and late complications and the long-term functional outcome and quality of life in patients who underwent restorative proctocolectomy with IPAA for UC and FAP patients during the past 20 years of follow-up. | longitudinal, 1984-2004,  study specific  questions measuring postoperative symptoms; QoL including physical function, social function, emotional problems, general health | 28 | FAP |
| [24] van Lier, 2010 | The Netherlands | The first aim of this study was to compare QoL and psychological distress in PJS patients to the general population, and the second aim was to identify determinants of QoLand psychological distress. | cross sectional,  questionnaires: SF-36, HADS, illness perception questionnaire – revised (IPQ-R), cancer worry scale | 52 | PJS |
| [25] van Lier, 2012 | The Netherlands | Therefore, the aim of this study was to investigate the desire to have children in PJS patients, and their attitudes towards PND with the implication of pregnancy termination and towards PGD. | cross sectional,  questionnaires: the cancer worry scale, Illness Perception Questionnaire-Revised, single items | 52 | PJS |
| [26] Lillehei, 2010 | US | Our present study was designed to prospectively evaluate and compare HRQOL before surgery and approximately one year postoperatively. Standardized HRQOL measures were utilized by the patients themselves, and their parents when feasible. | Longitudinal,  pre surgery, one year post surgery.  questionnaires: Child Health Questionnaire Parent Form, SF-36 | 10 | FAP |
| [27] Michie, 2001 | UK/Australia | This paper reports two studies. A cross sectional study compares the psychological impact of predictive genetic testing in children and adults. To the authors’ knowledge, this is the first such study. A prospective study compares children’s psychological functioning before and at two time points following testing. | prospective study: before/two time points post genetic testing, pos/neg results  questionnaires: STAI, The children’s version of STAI, the Hospital Anxiety and Depression Scale, Impact of Events Scale, The Rutter Child Behaviour Scale, regrets - single items, The health orentation scale, Perceptions of illness - single items, psychological resources - single items, | 54 | FAP |
| [28] Mireskandari, 2009 | Australia | To explore in detail the psychosocial impact of either having familial adenomatous polyposis (FAP) or being at risk for FAP amongst young adults. | cross sectional,  in depth interviews | 11 | FAP |
| [29] Ortega-Deballon, 2009 | France | We describe here the long-term results for 3 patients who benefited from gastric pouch construction. | cross sectional,  questionnaires: Gastro-Intestinal Functional Outcome (GIFO), SF-36 | 2 | FAP, IPAA |
| [30] Osterfeld, 2008 | Germany | To prospectively assess QoL in patients with FAP before and during the first year after proctocolectomy and IPAA. A particular focus of interest relates to the personal experiences during the transient ileostomy period and to the process of adaptation to the functional changes in patients’ everyday life. | retrospective cohort,  1993-2000,  questionnaires: SF-36, The Oresland-Score, HADS, optimism and self-efficacy short form questionnaire, F-SozU-K - social support, five illness-specific questions; semi structured interview | 21 | FAP |
| [31] Ozdemir, 2013 | US | To evaluate long-term control of neoplasia in the AT Z after IPAA and to assess the influence of anastomotic technique (handsewn vs stapled) on these results. | retrospective cohort, 1983-2010;  questionnaire: Cleveland global quality-of-life score | 260 | FAP |
| [32] Pan, 2024 | China | The goal was to obtain a comprehensive understanding of the reproductive concerns and needs among PJS patients in this age group and to explore factors associated with promoting reproductive health, ultimately enhancing the reproductive experience of PJS patients of childbearing age. | cross-sectional,  individual interviews | 16 | PJS |
| [33] Parc, 2000 | US | The aim of this study was to assess the results of IPAA in a series of 48 teenagers with FAP with regard to postoperative complications, functional results, and impact on their quality of life. | retrospective cohort, post surgery, 1981-1998,  study specific questionnaires: measuring post operative symptoms and function, social life; quality of life | 48 | FAP |
| [34] Raviram, 2015 | India | To assess QoL, functional outcome and social impact in patients undergoing IPAA for UC or FAP | longitudinal: 1, 3, 5 years post surgery,  questionnaire: Cleveland Global Quality of Life (CGQL) score | 13 | FAP |
| [35] Schneider, 2015 | Germany | To report on the functional outcome and quality of life (QoL) following standardized construction of a shorter J-pouch with a limb of 8–9 cm length. | cross sectional,  questionnaire:  SF-36 | 46 | FAP |
| [36] Wolf, 2011 | Germany | To elucidate how patients perceive their HRQL in the long-term follow-up after RPC to measure the impact of functional outcome, medical and psychosocial factors on patient-reported HRQL, and finally to define the medical, functional and psychosocial aspects that characterize subgroups of patients with compromised HRQL. | cross sectional, long term 8 years,  questionnaires: SF-36, The Oresland score, Self-efﬁcacy, Optimism and PessimismScale, 14-item questionnaire of social support, study specific questions; interview structured | 116 | FAP |
| [37] Woo, 2009 | US | To develop a PJS quality of life questionnaire using expert opinions of 3 cancer genetic counselors and a survey of patients with PJS through recruitment of participants involved in a support group over the internet. | cross sectional,  questionnaires: Center for Epidemiologic Studies, Depression Scale (CES-D), SF-36 | 32 | PJS |
| [38] Wood, 2019 | US | We hypothesized that FAP is commonly associated with mental health symptoms and that sometimes these may lead to a PTSD-like illness. If these hypotheses are confirmed, the implications for patient care will be significant. | cross sectional,  study specific questionnaires measuring different mental health symptoms, quality of life | 79 | FAP |
| [39] de Zeeuw, 2011 | The Netherlands | To evaluate the long-term results of ileoneo- rectal anastomosis (INRA) for FAP in terms of function, morbidity and quality of life (QoL). | Retrospective, median follow up was 7 years (range 4–12 years).  questionnaires: World Health Organization Quality of Life assessment instrument (WHOQOL-100), RAND-36 | 8 | FAP |
